# Nonparametric Evaluation of Quantitative Traits in Population-Based Association Studies when the Genetic Model is Unknown

**DOI:** 10.1371/journal.pone.0031242

**Published:** 2012-02-21

**Authors:** Frank Konietschke, Ondrej Libiger, Ludwig A. Hothorn

**Affiliations:** 1 Department of Medical Statistics, University of Göttingen, Göttingen, Germany; 2 Scripps Genomic Medicine, The Scripps Research Institute, La Jolla, California, United States of America; 3 Institute of Biostatistics, Leibniz-University Hannover, Hannover, Germany; Queen's University Belfast, United Kingdom

## Abstract

Statistical association between a single nucleotide polymorphism (SNP) genotype and a quantitative trait in genome-wide association studies is usually assessed using a linear regression model, or, in the case of non-normally distributed trait values, using the Kruskal-Wallis test. While linear regression models assume an additive mode of inheritance via equi-distant genotype scores, Kruskal-Wallis test merely tests global differences in trait values associated with the three genotype groups. Both approaches thus exhibit suboptimal power when the underlying inheritance mode is dominant or recessive. Furthermore, these tests do not perform well in the common situations when only a few trait values are available in a rare genotype category (disbalance), or when the values associated with the three genotype categories exhibit unequal variance (variance heterogeneity). We propose a maximum test based on Marcus-type multiple contrast test for relative effect sizes. This test allows model-specific testing of either dominant, additive or recessive mode of inheritance, and it is robust against variance heterogeneity. We show how to obtain mode-specific simultaneous confidence intervals for the relative effect sizes to aid in interpreting the biological relevance of the results. Further, we discuss the use of a related all-pairwise comparisons contrast test with range preserving confidence intervals as an alternative to Kruskal-Wallis heterogeneity test. We applied the proposed maximum test to the Bogalusa Heart Study dataset, and gained a remarkable increase in the power to detect association, particularly for rare genotypes. Our simulation study also demonstrated that the proposed non-parametric tests control family-wise error rate in the presence of non-normality and variance heterogeneity contrary to the standard parametric approaches. We provide a publicly available R library nparcomp that can be used to estimate simultaneous confidence intervals or compatible multiplicity-adjusted p-values associated with the proposed maximum test.

## Introduction

Genome-wide association studies involving large population-based samples have become a common strategy employed for the identification of common variants that affect a particular trait or play a role in disease. A majority of these studies involve comparing allele frequencies of di-allelic markers (e.g., SNPs) in cases and controls (see e.g., [1]). Formally, this is often accomplished via the Cochran-Armitage trend test [2] as implemented in the publicly available software PLINK [3]. Since the mode of inheritance at a given locus is often unknown, a maximum-test based on three mode-specific standardized Cochran Armitage trend tests was proposed [4].

Alternatively, continuous endpoints (i.e., quantitative traits), such as uromodulin [1] or TNFa protein [5] are commonly analyzed via a linear regression model using genotype scores 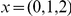 adjusted for covariates [6]. In order to maintain statistical validity, this approach requires that three important assumptions be met: 1. additive mode of inheritance; 2. normally distributed errors, and; 3. homogeneous variances. However, in reality one or more of these assumptions are often violated. The underlying mode of inheritance is often unknown. In addition, the assumption of normality is violated in studies that involve pQTL data [5], continuous endpoints with outliers (e.g., [6]), ordered categorical data (e.g., [7]), or phenotypes with values below the detection limit (e.g., [5]). Although transformation of the endpoints into an approximate normal distributed variable allows the use of standard approaches in the generalized linear model, the transformation is data-dependent, i.e. the choice of log-, log+constant, Box-Cox-transformation for pQTLs [6] might result in different conclusions. In particular, the re-transformation on the original scale is not unique. Nonparametric regression models, e.g. quantile regression [6], are an interesting option, however, up to now, only available for an additive mode of inheritance.

Nonparametric approaches do not require normality. However, the often used nonparametric Kruskal-Wallis test [1],[8],[9] achieves suboptimal power when the locus is governed by a specific mode of inheritance. This occurs because it is a global test of heterogeneity in the endpoint values among the three genotype groups. It is also not robust against variance heterogeneity. Jonckheere-Tepstra test [10], an analog of the Kruskal-Wallis test for near-to-linear ordered restricted alternatives, shares many characteristics with the Kruskal-Wallis test, while being particularly sensitive to an additive mode of inheritance. Furthermore, Kruskal-Wallis and Jonckheere-Tepstra nonparametric procedures are global testing procedures based on global ranks whose distribution is only available under the global null hypothesis. Therefore, it is not possible to compute confidence intervals for the genetic effects of interest using these approaches. In summary, none of these classic nonparametric approaches i) allow the identification of the most likely mode of inheritance via estimation of related simultaneous confidence intervals, ii) are sensitive not only to an additive mode of inheritance, or iii) are robust against variance heterogeneity. Our proposed testing procedure, on the other hand, can be extended to provide this crucial information for interpreting the biological relevance of the association results.

Recommendations given in the ‘Strengthening the Reporting of Genetic Association studies’ report [11] include providing estimators of an adequate effect size and their confidence intervals. For example, reporting odds ratios for additive, recessive and dominant models and their marginal confidence limits (as in e.g., [12]) provides a percentage measure of clinical relevance (distance from the lower/upper confidence limit to one, the value associated with the null hypothesis). While traditional significance testing usually deals with differences between population means, there is an increasing focus in medicine on the probability of one treatment being more successful than another on a per-individual basis [13]. The relative effect size [14]

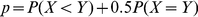
(1)represents *a measure of how often a randomly chosen subject receiving treatment X will outperform a randomly chosen subject receiving treatment Y* [13], i.e. the probability that a randomly selected subject in the control reveals a smaller response value than a randomly selected subject in the treatment group. In case of ordered categorical data, 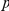 is also called *ordinal effect size measure* [15].

We describe a Behrens-Fisher version of multiple contrast test for relative effects [16],[14] based on the maximum test principle [4]. This is a purely nonparametric testing procedure that is valid when the three assumptions mentioned in the previous paragraph are not met. Furthermore, our proposed approach simultaneously tests association under the assumption of the three basic modes of inheritance, additive (add), recessive (rec) and dominant (dom) for both continuous and discontinuous distributions. We generalize the relative effect p for an adequate formulation of genetic effects, and provide multiple contrast tests and simultaneous confidence intervals, which allow the simultaneous testing of the three genetic models of inheritance.

## Methods

### Motivating example

**Figure 1:**
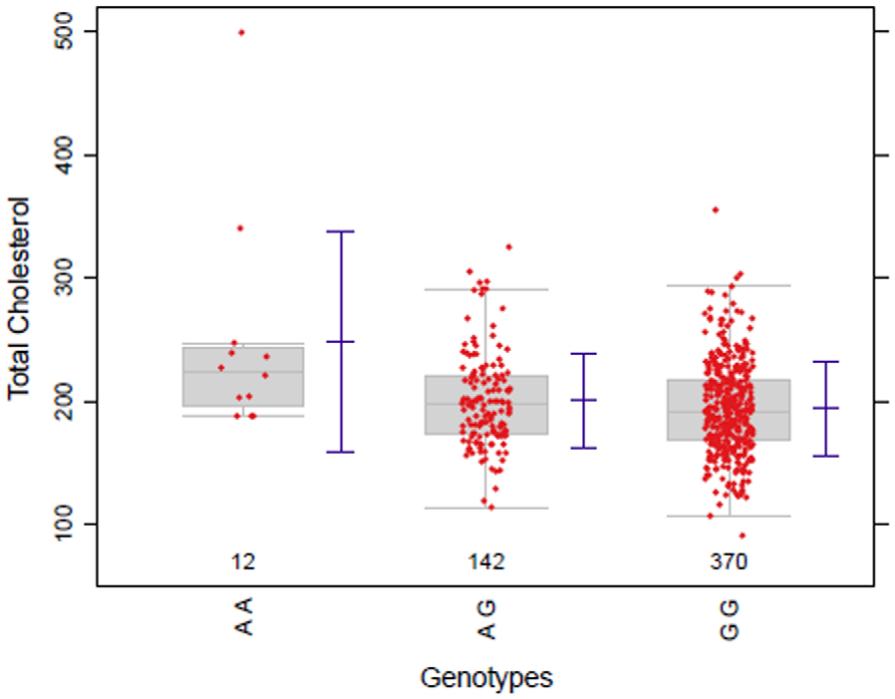
Boxplot: Total cholesterol vs. genotype rs7738656.

A real data example with the right skewed distributed phenotype *total cholesterol* was selected from the Bogalusa Heart Study (BHS) [17]. This longitudinal study included genotype information on 525 unrelated individuals of European descent at 545,821 SNPs where twelve clinically-relevant quantitative traits were measured for each study participant. We applied the nonparametric multiple contrast test to a one-way layout for SNP *rs7738656* in the gene C6orf170/GJA1 and the phenotype *total cholesterol*, which was published in Table 1 of the original paper as significant for an unimputed SNP [18]. The jittered boxplots in Figure 1 show an unbalanced design, variance heterogeneity and a rather skewed distribution with some extreme values, particularly for the homozygote minor allele genotype group. Therefore, the question arises whether the parametric analysis in the original publication using a linear regression model assuming an additive mode of inheritance and normally distributed errors with homogeneous variances is appropriate.

**Table 1:** 95-%Simultaneous confidence intervals for 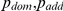 and 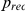 for the SNP rs7738656.

Model	Effect-Estimator	95%-Simultaneous Intervals	Adjusted p-Value
Dominant	−0.240	[−0.378; −0.082]	0.0058
Additive	−0.250	[−0.387; −0.092]	0.0043
Recessive	−0.053	[−0.119; 0.015]	0.13

### Nonparametric model and genetic effects

Let 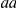, 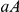 and 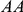 denote the genotypes, where 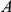 is the high risk allele and 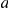 is any of the other alleles. For convenience, abbreviate the genotypes with 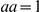, 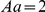, and 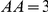. The related data are given by 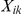, where 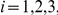 and 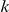 denotes the subject within genotype level 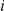, 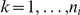. The data 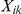 are assumed to be independent. The total sample size is 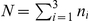. We assume that the phenotypes 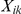 follow an arbitrary distribution 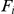, i.e.
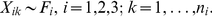
(2)


This general model (2) does not contain any parameters that could be used to describe a difference between the distributions. Therefore, the distribution functions 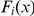 are used to define purely nonparametric treatment effects on an individual basis for each genotype level by
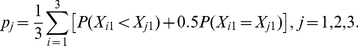
(3)These effects are also called unweighted relative effects [19], [20]. If 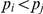, then the values from 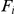 tend to be smaller than those from 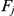. In case of 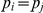, none of the observations tend to be smaller or larger. Therefore, these effects can be as easily interpreted as the usual means in parametric models. Let 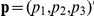 denote the vector of the unweighted relative effects.

For the formulation of nonparametric genetic effects, let
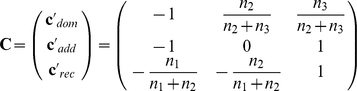
denote the so-called Marcus-type contrast matrix [21]. In case of a balanced design, 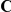 reduces to
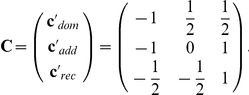
Each row vector 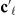 of 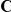 corresponds to one of the three genetic models. In case of a dominant mode of inheritance, the distributions 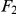 and 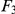 are identical, therefore a relative genetic effect for this mode can be expressed by
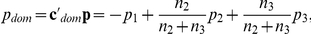
which denotes the difference between the pooled effect 
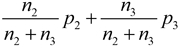
 among the samples 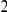 and 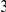 and 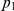. Thus, in case of “no dominant effect”, 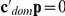, or equivalently, 
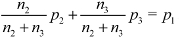
. Analogously, in case of a recessive mode of inheritance, the distributions 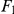 and 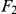 are identical, thus, a relative recessive effect can be expressed by
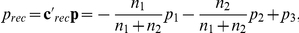
which denotes the difference between the pooled effect 
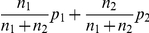
 and 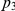. “No recessive mode of inheritance” means 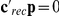, or, equivalently, 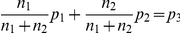. In addition, the relative genetic effect for an additive mode of inheritance can be expressed by
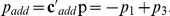



Thus, the case of *no global effect* is characterized by 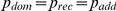, or, equivalently, 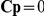.

### A multiple contrast test approach for the three genetic models

To test the individual hypothesis 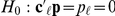, where 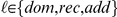, define a test statistic 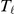, which denotes, as usual, a studentized estimator 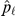 of 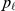 with its estimated standard error (details see Technical details). The three test statistics 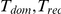 and 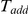 are collected in the vector
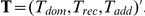
The multiple contrast test and the simultaneous confidence intervals for 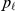 are based on the asymptotic multivariate normality of 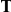, i.e. the correlation among the three test statistics 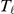 is accounted for. Instead of using critical values coming from a standard normal distribution (or t-distribution), we use critical values from the multivariate normal distribution 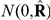, where 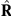 denotes the estimated correlation matrix. This means, the individual hypothesis 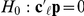 is rejected at multiple level 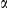 of significance, if
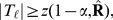
(4)where 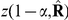 denotes the 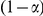-equicoordinate quantile from 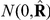. Simultaneous confidence intervals for the three genetic effects 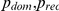 and 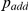 are given by
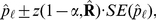
(5)i.e. point estimator 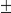 quantile 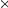 estimated standard error. Note that the individual test decisions and the simultaneous confidence intervals are compatible, i.e. it can not occur that an individual hypothesis has been rejected, but the corresponding simultaneous confidence interval includes the value from the null hypothesis. These confidence intervals, however, may not be range preserving, i.e. the lower bounds may be smaller than 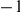 and the upper bounds can be larger than 1. Range preserving confidence intervals can be easily constructed by using the delta method [16], [22], with the Fisher transformation. The global hypothesis 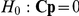 will be rejected, if
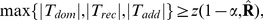
(6)i.e. if any of the three individual hypotheses have been rejected. For small sample sizes, the quantiles from the multivariate normal distribution are replaced by quantiles coming from a multivariate t-distribution (details see Technical details).

#### Evaluation of score phenotypes

Particularly in psychiatric epidemiology, different mental scores are often used as phenotypes, see e.g. [7]. Some mental scores are based on only few categories, e.g. 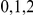, others represent sums of sub-scores with a wider range of count values. The definition of the unweighted relative effect 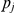 defined in (3) includes ordered categorical data. For an arbitrary monotone transformation 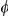 of the data, it can be seen that
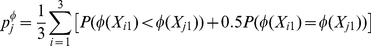


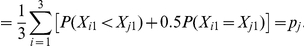
Thus, the effect measure is invariant under monotone transformations of the data. On the other hand, if the data are transformed by a monotonic decreasing function 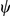


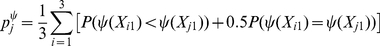


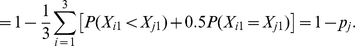
This means that the effect measure 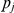 is reflected at 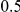 in case of a monotonic decreasing transformation of the data. Therefore, 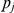 is an adequate measure for ordered categorical data, because the information is independent from the chosen scale of the scores.

#### Evaluation of phenotypes with values below a detection limit

Sometimes phenotypes with values below a detection limit occur, see e.g. [5]. Since the ordinal effect size is appropriate for tied values, all data below the detection limit should be fixed on a particular value. Note that this approach is only exact when a unique detection limit exists, i.e. the problem is more complex when different centers in a meta-analysis framework have different detection limits. However, due to the ranking of the groups, the problem of choosing the “best value” will not occur.

## Results

### Evaluation of the example

The new nonparametric multiple contrast test 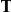 was used for the statistical analysis of the motivating example above. Rank estimators for the three unweighted relative effects are given by 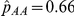, 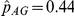 and 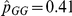, respectively. Assuming *AA* is the risk allele, a decrease to *AG* and *GG* occur. Table 1 summarizes the results of simultaneous Marcus-type comparisons.

The upper confidence limit of the additive model is most distant to 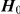, or, compatible to that, reveals the smallest p-value of 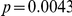. The 95%-simultaneous confidence intervals indicate a positive association with the high risk allele 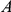 for the phenotype *total cholesterol*. The related parametric approach results a much smaller p-value of 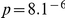 for the additive mode of inheritance (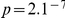 in the original publication [17] with an adjustment against covariates). This example illustrates the impact of the underlying assumptions being violated, in particular the assumption of normally distributed errors with homogeneous variances. The global rank Kruskal-Wallis test on heterogeneity reveals a p-value of only 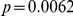.

In summary, using the multiple contrast tests yields specific information regarding the genetic mode of inheritance as well as simultaneous confidence intervals.

### Simulations

We evaluated the empirical type-I error rates and the powers of nonparametric multiple contrast tests via extensive simulation studies. All simulations were performed using the publicly available software R (version 2.12.1; www.r-project.org). Every simulation step was repeated 10,000 times.

**Figure 2:**
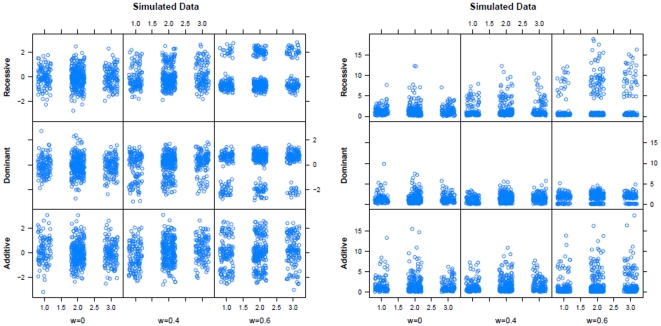
Simulated normal (left) and log-normal (right) data for different values of variance explained by the quantitative trait 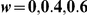
 **,** 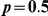 **,** 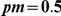 **,** 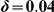 **and an additive, dominant and recessive mode of inheritance.**

The trait genotypes for 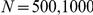 subjects were randomly drawn from a multinomial distribution with cell probabilities given by allele frequencies at trait locus 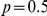, allele frequencies at trait marker 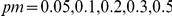 and linkage disequilibrium delta 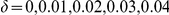. Phenotypic values for the quantitative traits were generated from normal and log-normal distributions, choosing 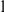 for the residual variance, and varying the percentage of variance explained by the quantitative trait 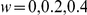 for an additive, dominant, or recessive mode of inheritance. Log-normal phenotypes were generated by first drawing normal phenotypic values 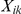 and then by applying the transformation method 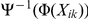, where 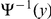 denotes the quantile function of the log-normal distribution, and 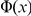 denotes the standard normal distribution function. If 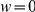, no variance is explained by the quantitative trait, thus, 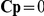 for all parameter settings. Low values of allele frequencies at trait marker (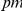) result in strongly unbalanced designs. In addition, different values of 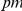, 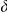, and 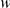 form specific multimodal distributions under the alternative. Figure 2 displays examples of simulated normal and log-normal data for different values of 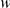, 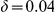, 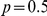 and 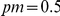.

Since the expectation of a multimodal distribution is the weighted sum of the single expectations, the parameter settings on 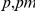 and 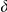 are an important issue in the investigation of power analyses.

#### Results

**Figure 3:**
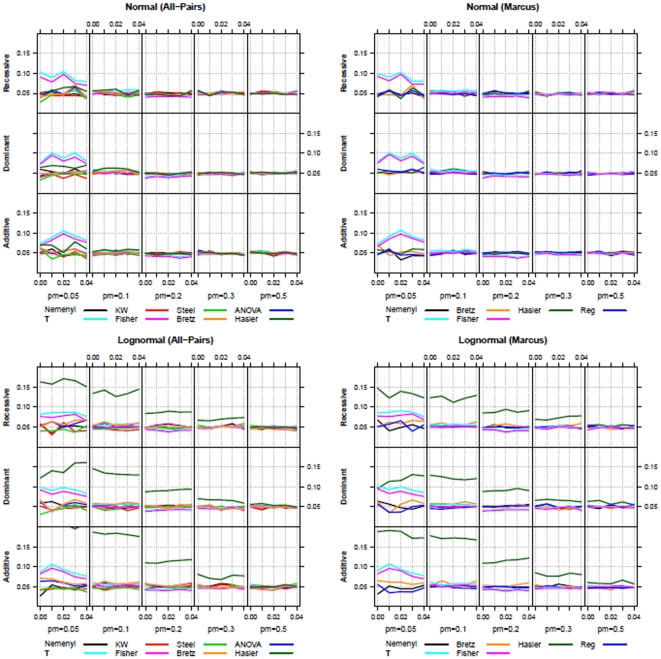
Type-I error ( 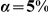
 **) simulation results for all-pairs (left) and Marcus-type (right) comparisons using normal (upper row) and log-normal (lower row) distributions (** 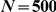 **).**

We simulated the nonparametric multiple contrast tests 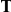 as defined in (6) as well as its transformed approach by using the Fisher-transformation (*Fisher*). Two different types of contrasts will be examined throughout the simulation studies: (i) all-pairwise comparisons by using the contrast matrix
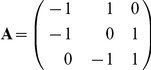
to be sensitive against any heterogeneity (*All-Pairs*) and (ii) the Marcus-type contrast matrix 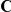 to be sensitive against exactly the three basic genetic modes of inheritance (*Marcus*). For each kind of contrast, the nonparametric multiple contrast tests are compared with the parametric multiple contrast tests for homoscedastic normal samples proposed by [23] as well as for heteroscedastic normal samples by [24] (denoted by *Bretz* and *Hasler*). For all-pairs comparisons, these four multiple contrast test procedures are compared with the nonparametric multiple test procedures by Steel [25] (*Steel*), the permutative Nemenyi-test [26] for all-pairs comparisons (*Nemenyi*), the Kruskal-Wallis test (*KW*) and the usual ANOVA-F-test (*ANOVA*). For Marcus-type comparisons, the four multiple contrast tests are compared with the nonparametric permutative Nemenyi-test for Marcus-type comparisons and the usual linear regression analysis (*Reg*). Figure 3 displays the type-I error simulation results (i.e. 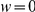; 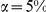) for different values of 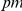, an additive, dominant, and recessive mode of inheritance, and linkage disequilibrium delta 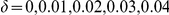 for both normal and log-normal distributed phenotypes (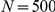).

It follows from Figure 3, that, under normality, all considered procedures control the type-I error at level 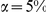 for both *all-pairs* and *Marcus*-type comparisons. In the case of extremely unbalanced designs (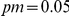), however, the new multiple contrast tests 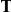 and *Fisher* tend to be quite liberal. This is due to the fact that these procedures do not use a pooled variance estimator. We observed this for both normally and log-normally distributed data. In the case of larger sample sizes (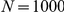), this effect disappears. The parametric multiple contrast test by *Hasler* tends to be very liberal when the normality assumption is violated.

**Figure 4:**
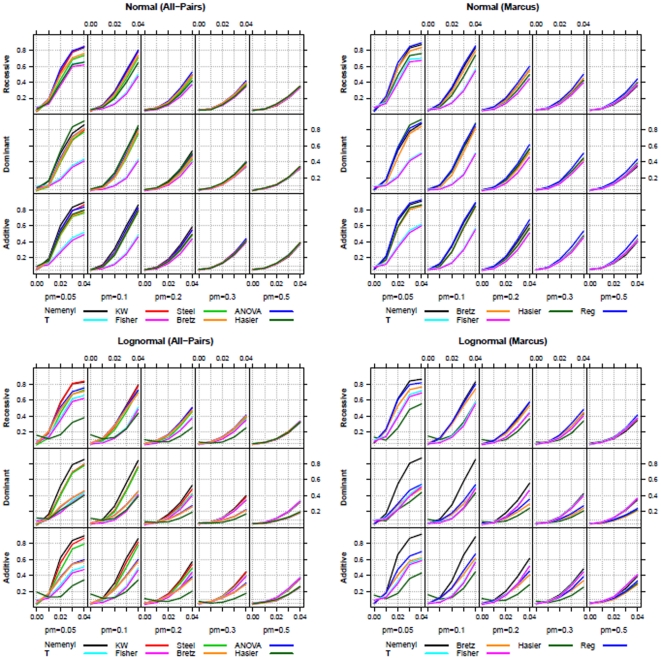
Power-simulation results ( 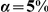
 **) for all-pairs (left) and Marcus-type (right) comparisons using normal (upper row) and log-normal (lower row) distributions (** 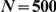 **).** The variance explained by the quantitative trait was set to 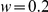.

To investigate the power of the different procedures mentioned above, different parameter settings on the variance explained by the quantitative trait (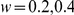) and different values on 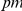 and 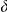 were examined. Figure 4 displays the simulation results for 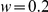.

**Figure 5:**
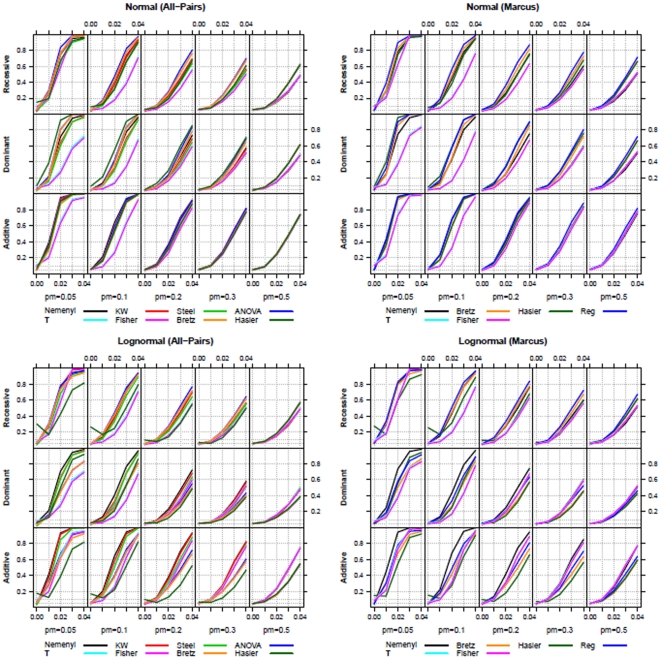
Power-simulation results ( 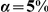
 **) for all-pairs (left) and Marcus-type (right) comparisons using normal (upper row) and log-normal (lower row) distributions (** 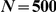 **).** The variance explained by the quantitative trait was set to 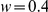.

Figure 4 shows that the power of all investigated procedures depends on the parameter settings of 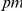 and 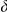. The combination of these parameters leads to specific values of weights in the multimodal distributions of the phenotypes as displayed in Figure 2. We observe that for a given 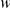 and 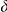, the power of the tests is smaller for larger 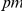, although the data are almost balanced in such settings. This occurs, because the weighting parameters of the multimodal distributions are likely in case of smaller allele frequencies at trait marker. In case of 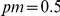, the bimodal distributions consist of a dominated and a dominating part, which results in a smaller expectation of the phenotypic values in all considered cases. In case of extremely unbalanced designs (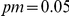), the power of the new procedures is quite low; in general, their power is not estimable due to their liberality in such settings. For normal distributions, the powers of all the parametric and nonparametric procedures are nearly identical in case of 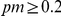. When the normality assumption is violated, the nonparametric procedures have a considerably higher power than the parametric procedures. The power of the new multiple contrast tests are likely to be identical to the power of the Kruskal-Wallis test. The Kruskal-Wallis test, however, can only be used for testing the global null hypothesis, and cannot provide any information regarding genetic association. Further, comparing the results of the all-pairs and Marcus-type comparisons, we observe that all the four multiple contrast tests exhibit higher power when using the Marcus-type contrast matrix compared to using the Tukey-type contrast matrix 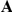. Simple linear regression analysis should not be used, because (i) the genotypic values are not metric numbers and thus the results depend on the chosen numbers for the three genotype scores and (ii), in all simulations the regression does not provide a considerably higher power than the multiple contrast test procedures. The same conclusions can be drawn for the simulation results obtained by 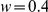, which are displayed in Figure 5.

### Software

For a convenient application of the developed procedures, the R-software package *nparcomp* was developed and is available from CRAN. It contains various functions for the analysis of two independent samples (*npar.t.test*), as well as functions for the computation of nonparametric multiple contrast tests and simultaneous confidence intervals based on global ranks and pairwise ranks. For example, the function *nparcomp* computes simultaneous confidence intervals and adjusted p-values for relative effects in arbitrary contrast settings based on pairwise ranks. Moreover, one-sided and two-sided confidence intervals and adjusted p-values are computed using multivariate normal-approximation, multivariate 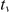-approximation, Logit-approximation and Probit-approximation described in [16].

## Discussion

A nonparametric approach to evaluate the association between a di-allelic marker and a non-normal distributed quantitative trait is proposed for simple population-based studies. Using a Marcus-type multiple contrast test for relative effects allows model-specific testing of either dominant, additive or recessive mode of inheritance. Furthermore, an all-pairwise comparisons contrast test is proposed as an alternative to the Kruskal-Wallis heterogeneity test. Procedures for obtaining related simultaneous confidence intervals or multiplicity-adjusted p-values are provided. The advantage of obtaining confidence intervals is their interpretability in terms of stochastic order for studies with individuals according to [13]. Although related software is freely available using the R library nparcomp, the routine analysis of hundreds of thousands of SNPs can not be recommended. The computing time would be excessive and the amount of detailed information difficult to manage. For some selected candidate SNPs this approach can be easily performed for a number of phenotypes. If still an analysis on a genome-wide level is intended, an appropriate multiplicity adjustment of the simultaneous confidence is recommended, such as the false coverage statement rate [27].

Adjustment against multiple covariates is an important issue in unbiased testing association. The adjustment against population stratification, e.g. by principle components [28], or subject-specific baseline values, e.g. age, are relevant. For example [6] adjusted the relationship between an eQTL and the genotype scores against the covariates age, kind of tissue (kidney cortex or medulla), ancestry (CEU or not) and gender (males or females). Nonparametric analysis of covariance is challenging [29], particularly to adjust against covariates due to possible population stratification. This is a topic of future work.

### Technical details

To estimate the unknown relative effect 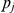 defined in (3), let
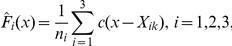
denote the empirical distribution function, where 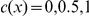 according to 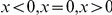, respectively. An unbiased estimator of 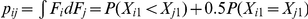 as used in (3) is obtained by replacing the unknown distributions 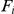 and 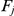 by their empirical counterparts 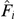 and 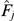. The estimators
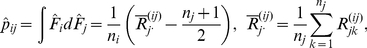
(7)can be easily computed with the ranks 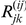 of the observations 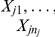. Here, 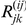 denotes the rank of 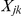 among all 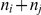 observations in the combined sample 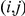. Thus, an estimator of 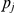 is given by
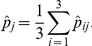
The ranks used for the estimation of 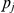 are also called *pseudo-ranks* in the literature [20]. In case of a balanced design (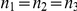), the pseudo-ranks are identical to the usual global ranks. Let 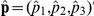 denote the vector of the three estimators. Thus, rank estimators of the three relative genetic effects 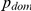, 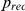, and 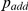 are given by 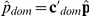, 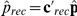, and 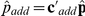, respectively.

It was shown that 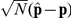 is asymptotically multivariate normal with mean 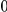 and covariance matrix 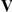 [16], [22]. Due to the quite involved structure of 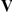, let the estimator of 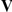 be denoted by 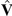 [16], [22]. To test each individual hypothesis 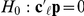 on no genetic association, where 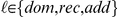, let 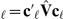 denote the variance estimator of 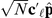 and define the test statistic
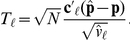
The three test statistics 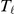 are collected in the vector
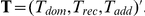
The distribution of 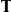 can be approximated by a multivariate 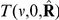 distribution, where 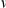 denotes a Welch-Satterthwaite degree of freedom, non-centrality vector 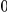, and estimated correlation matrix 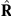 [16]. The individual hypothesis 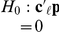 will be rejected at multiple level 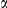, if
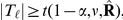
where 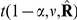 denotes the 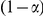 equicoordinate quantile of 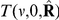. Approximate 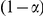 simultaneous confidence intervals for the three genetic effects 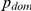, 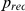, and 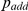 are obtained from
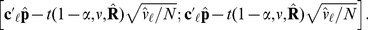
(8)The global hypothesis 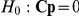 will be rejected, if
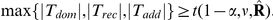
Range preserving confidence intervals are given by
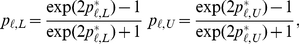
(9)where
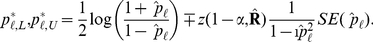
(10)


Alternatively, a pairwise rankings version is available which can be easily derived from two-sample tests. They behave similarly to the global rankings approach, but they can lead to paradoxical results.

### Pairwise rankings version

As mentioned in the previous section, the three genetic effects 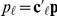, 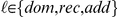, denote generalized two-sample relative effects, which were estimated with global ranks of the data 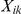. Thus, the effects can be modified such that pairwise ranks are used for estimation. Let
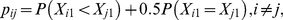
(11)denote the two-sample relative effect between the genotype levels 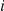 and 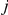. If 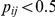, then the values from 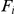 tend to be larger than those from 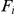. In case of 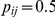, none of the observations tend to be smaller or larger. Thus, the case of *no association* can be expressed by 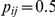. The relative dominant genetic effect on association describes the difference between the distribution 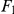 and the combined sample 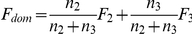. Thus, a two-sample relative dominant effect can be described by
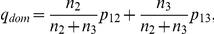
and denotes a linear combination of 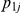. The relative recessive effect describes the difference between the combined sample 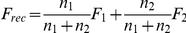 and 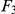. Thus, a relative effect on a recessive mode of inheritance is given by
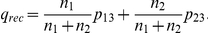
Finally, the relative two-sample effect on an additive mode of inheritance can be expressed by
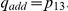
The effects 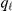 can be estimated by using the pairwise rank estimators 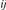 defined in (7) by
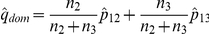


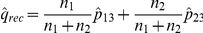


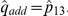
Multiple contrast tests for the hypotheses 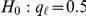 and simultaneous confidence intervals for the effects 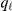, where 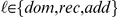, can be derived in the same way as described in the previous section. We note that the effects 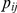 may be intransitive, i.e. it may occur that 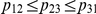 resulting in paradoxical results [30], [31]. Therefore, we recommend using the global ranking version.
